# Advances in CRISPR/Cas-based Gene Therapy in Human Genetic Diseases

**DOI:** 10.7150/thno.43360

**Published:** 2020-03-15

**Authors:** Shao-Shuai Wu, Qing-Cui Li, Chang-Qing Yin, Wen Xue, Chun-Qing Song

**Affiliations:** 1 Key Laboratory of Growth Regulation and Transformation Research of Zhejiang Province, School of Life Sciences, Westlake University, 18 Shilongshan Road, Hangzhou 310024, Zhejiang Province, China.; 2 RNA Therapeutics Institute, University of Massachusetts Medical School, Worcester, Massachusetts; 3 Program in Molecular Medicine and Department of Molecular, Cell and Cancer Biology, University of Massachusetts Medical School, Worcester, Massachusetts

**Keywords:** CRISPR/Cas, Gene editing, Gene therapy, Human disease, Genetic disease

## Abstract

CRISPR/Cas genome editing is a simple, cost effective, and highly specific technique for introducing genetic variations. In mammalian cells, CRISPR/Cas can facilitate non-homologous end joining, homology- directed repair, and single-base exchanges. Cas9/Cas12a nuclease, dCas9 transcriptional regulators, base editors, PRIME editors and RNA editing tools are widely used in basic research. Currently, a variety of CRISPR/Cas-based therapeutics are being investigated in clinical trials. Among many new findings that have advanced the field, we highlight a few recent advances that are relevant to CRISPR/Cas-based gene therapies for monogenic human genetic diseases.

## Introduction

The past 20 years have witnessed great progress in genome editing techniques, including meganucleases, zinc finger nucleases (ZFN), transcription activator-like effector nucleases (TALENs), and the clustered regularly interspaced short palindromic repeats (CRISPR) and CRISPR-associated (Cas) nuclease system. These tools hold great potential for treating human disease, especially genetic diseases beyond the reach of traditional approaches 1. The CRISPR/Cas system has rapidly become the most popular genome editing platform due to its simplicity and adaptability 2-5.

**Figure 1:**
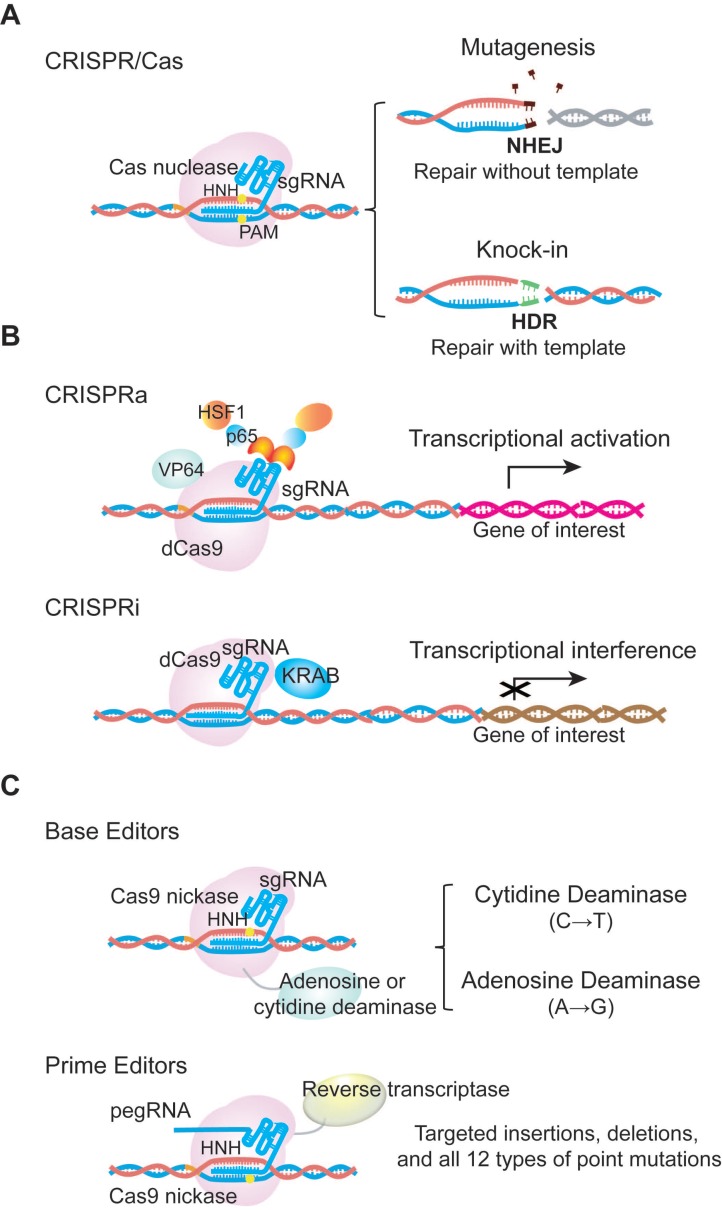
** Examples of CRISPR/Cas9 technological advances. (a)** Cas9 is directed by single guide RNA (sgRNA) to the target sequence. Double stranded DNA breaks are subsequently repaired by cellular DNA repair machinery via the NHEJ or HDR pathway. **(b)** dCas9 fused with transcriptional activators or repressors activates or inhibits the expression of a target gene. These systems are called CRISPRa or CRISPRi. dCas9 indicates catalytically inactive dead Cas9, which is able to bind the target DNA without cutting. CRISPRa, CRISPR activators to activate transcriptional process; CRISPRi, CRISPR inhibitors to interference transcriptional process. **(c)** Base editors are the combination of Cas9 D10A nickase with cytidine or adenine deaminase to induce G->T or A->G transition. Prime editor, different from base editors, is the fusion protein of Cas9 H840A nickase and reverse transcriptase. It can achieve up to 12 types of base-to-base conversions, and targeted insertions and deletions without DSBs or donor DNA templates. pegRNA, prime editing guide RNA.

The CRISPR/Cas system was originally discovered as a prokaryotic adaptive immunity system used to recognize and cleave invading nucleic acids 6-8. Based on this prokaryotic system, scientists have engineered a series of CRISPR/Cas tools for genome editing in mammalian cells, with the list of CRISPR/Cas systems in use continuing to expand. The most commonly used Cas nuclease comes from *Streptococcus pyogenes* (SpCas9), and belongs to the type II CRISPR system. SpCas9 was the first to be reprogrammed for genome editing in mammalian cells. For specific nucleotide sequence recognition, engineered SpCas9 relies on the guidance of a single-guide RNA (sgRNA). Typically, sgRNA is composed of a scaffold sequence that is bound by the Cas protein, and a custom-designed ∼20 nucleotide spacer that defines the genomic target to be modified. Following hybridization of the spacer to a target genomic sequence that is positioned next to a protospacer adjacent motif (PAM), the target DNA is cleaved, leading to a double-strand break (DSB) 7-9. The Cas-mediated DSB is subsequently repaired by cellular DNA repair machinery via homology- directed repair (HDR) or the non-homologous end joining (NHEJ) pathway. NHEJ can be used to produce insertions and deletions (indels) that disrupt or inactivate the target gene, while HDR can be used for precise nucleotide sequence modifications, such as point mutation correction 10-12 (**Figure 1a**).

To date, CRISPR/Cas-based techniques have been applied in various cell types and organisms. For therapeutic genome editing to treat monogenic diseases, CRISPR has the potential to be used directly in patients (*in vivo*) or in human cells (*in vitro*). In this review, we focus on CRISPR strategies used to treat human monogenic diseases, and discuss the challenges associated with these approaches.

## Recent advances in CRISPR/Cas technology

Shortly after SpCas9 was applied in mammalian cells, other Cas9 proteins have been studied and developed as genome editing tools. For example, smaller Cas9 proteins derived from *Staphylococcus aureus* called SaCas9 13 and *Neisseria meningitidis* called Nme2Cas9 14 exhibit gene editing efficiency comparable to that of SpCas9. These smaller Cas9s are more amenable for *in vivo* delivery than the large SpCas9 (~4.3 kb).

CRISPR/Cas9 technological advances have also enabled various applications of nuclease-deficient Cas9s, which can bind a specific region of the genome without creating DSBs. For example, catalytically inactive dead Cas9 (dCas9) can be fused with various transcription regulatory domains to create CRISPR activators (CRISPRa) or inhibitors (CRISPRi) that activate or silence the expression of a target gene 15 (**Figure 1b**). dCas9 can also be used as a visualization tool. Chen and colleagues have used dCas9 fused to enhanced green fluorescent protein (EGFP) to visualize repetitive DNA sequences using one sgRNA, or nonrepetitive loci using multiple sgRNAs 16-18. In addition, David R. Liu's group has fused D10A Cas9 nickase with either cytidine or adenine deaminase to generate cytidine base editors (CBEs) and adenine base editors (ABEs), respectively. CBEs and ABEs generate transitions between A•T and C•G base pairs without causing high levels of double-stranded DNA cleavage in the target genomic region. Importantly, the Liu's group has extended base editing to utilize H840A Cas9 nickase fused with reverse transcriptase to create prime editors (PEs), which can achieve all possible base-to-base conversions (12 in total), as well as targeted insertions and deletions without DSBs or donor DNA templates 19 (**Figure 1c**).

In addition to DNA editing, Feng Zhang's lab has reported that an RNA-targeting CRISPR system based on Cas13 can target and cleave specific strands of RNA, and subsequently developed strategies called REPAIR (RNA Editing for Programmable A to I Replacement) and RESCUE (RNA Editing for Specific C to U Exchange) to edit RNA 20, 21. Thus, RNA editing with CRISPR can efficiently modulate target genes at the transcript level in a transient and PAM independent manner. This approach could provide a controllable approach for disease treatment.

## Applications of CRISPR in genetic diseases

To date, CRISPR/Cas systems have been used to investigate target genes in genome modification 22, splicing 23, transcription 24 and epigenetic regulation 25, and have been applied in a research setting to investigate and treat genetic diseases 26, infectious diseases 27, cancers 28, and immunological diseases 29, 30. Among the exciting advances, translational use of CRISPR/Cas in monogenic human genetic diseases has the potential to provide long-term therapy after a single treatment. In this section, we summarize the recent applications of the CRISPR/Cas system in the generation of disease models and in the treatment of genetic diseases *in vitro* and *in vivo*.

### Disease modeling using CRISPR/Cas

The generation of disease models is necessary for understanding disease mechanisms and developing new therapeutic strategies. CRISPR/Cas has been widely used for creating disease-related cellular models, such as DMD 31, aniridia-related keratopathy (ARK) 32, brittle bone 33, X-linked adrenoleukodystrophy (X-ALD) 34, and Alzheimer's disease 35. Moreover, researchers have created a series of mouse models using CRISPR/Cas that recapitulate DMD 36, atherosclerosis 37, obesity and diabetes 38, RTHα 39, and Alzheimer's disease 40 (**Table 1**). One example is the development of a mouse model for ryanodine receptor type I-related myopathies (RYR1 RM), which harbors a patient- relevant point mutation (T4706M) engineered into one allele, and a 16-base pair frameshift deletion engineered into the second allele of the RYR1 gene. Subsequent experiments demonstrated that this mouse model of RYR1 RM is a powerful tool for understanding the pathogenesis of recessive RYR1 RM, and for preclinical testing of therapeutic efficacy 41. CRISPR/Cas has also been used to generate disease models in large animals, including sheep 42, rabbit 43, pig 44, and monkey 45. For example, a monkey model was developed to study Parkinson's disease by introducing a *PINK1* deletion and revealed a requirement for functional PINK1 in the developing primate brain 45. CRISPR/Cas technology offers a flexible and user-friendly means of developing disease models to explore the genetic causes of diseases and evaluate therapeutic strategies.

### Disease correction using CRISPR/Cas in model organisms and clinical trials

Monogenic diseases affect a large population of patients. In the ClinVar database, more than 75,000 pathogenic genetic variants have been identified 19, 46. Here we summarize recent therapeutic applications of CRISPR/Cas in model organisms and in clinical trials (**Table 2 and Table 3**).

### Hemoglobinopathies

Inherited blood disorders are good candidates for gene therapies because gene therapy can modify the causative gene in autologous hematopoietic stem cells (HSCs) and correct the hematopoietic system. β-thalassemia and sickle cell disease are two genetic blood diseases. β-thalassemia is due to various mutations including small insertions, single point mutations or deletions in β-globin gene, resulting in loss or reduced β-globin synthesis 47. Sickle cell disease is caused by a Glu->Val mutation in β-globin subunit of hemoglobin 48, 49, leading to abnormal hemoglobin S. Re-expressing the paralogous γ-globin genes is a universal strategy to ameliorate both β-globin disorders. The Bauer group applied CRISPR/Cas-based cleavage of the GATA1 binding site of the erythroid enhancer. This approach decreases erythroid expression of the γ-globin repressor BCL11A and in turn increases γ-globin expression. This strategy is therapeutically practicable to produce durable fetal hemoglobin induction 50-52 (**Table 2**).

To date, three clinical trials aiming to treat patients with β-thalassemia and severe sickle cell disease by transfusion of CRIPSR/Cas9 edited CD34+ human HSCs (CTX001) have been initiated by CRISPR Therapeutics in 2018 and Allife Medical Science and Technology Co., Ltd in 2019 (**Table 3**).

### Inherited eye disease

Leber congenital amaurosis (LCA) is a rare genetic eye disease manifesting severe vision loss at birth or infancy. LCA10 caused by mutations in the *CEP290* gene is a severe retinal dystrophy. *CEP290* gene (~7.5 kb) is too large to be packaged into a single AAV. To overcome this limitation, Editas Medicine developed EDIT-101, a candidate genome editing therapeutic, to correct the *CEP290* splicing defect in human cells and in humanized CEP290 mice by subretinal delivery. This approach uses SaCas9 to remove the aberrant splice donor generated by the IVS26 mutation. In the human CEP290 IVS26 knock-in mouse model, over 94% of the treated eyes achieved therapeutic target editing level (10%) when the dose of AAV was not less than 1 × 10^12^ vg/ml 53. Allergan and Editas Medicine have initiated a clinical trial of EDIT-101 for the treatment of LCA10 (**Table 3**).

Autosomal dominant cone-rod dystrophy (CORD6) is induced by a gain-of-function *GUCY2D* mutation. CRISPR/Cas components delivered by AAV specifically disrupt the early coding sequence of *GUCY2D* in the photoreceptors of mice and macaques by NHEJ. This study was the first to successfully perform somatic gene editing in primates using AAV-delivered CRISPR/Cas (up to 13% editing efficiency of *GUCY2D* mutant gene in macaque photoreceptor), and demonstrated the potential of CRISPR/Cas to cure inherited retinal diseases 54.

### Muscular genetic disease

DMD, caused by mutations in the dystrophin gene, is the most common form of progressive muscular dystrophy, and is characterized by muscle weakness, loss of ambulation, and premature death. Several groups have used NHEJ to bypass a premature stop codon in exon 23 and restore the expression of dystrophin in neonatal and adult mice after local or systemic delivery of CRISPR/Cas components by AAV 55-57. Similarly, CRISPR/Cas- induced NHEJ has been used to treat DMD in a DMD dog model after AAV-mediated systemic delivery of CRISPR gene editing components. 3 to 90% of dystrophin was recovered at 8 weeks after systemic delivery in skeletal muscle, the editing efficiency was dependent on muscle type and the muscle histology was improved in treated dogs 58. In addition, ABE was delivered locally by intramuscular injection of a trans-splicing AAV to cure DMD in a mouse model 59. These studies highlight the potential application of gene editing for the correction of DMD in patients.

Congenital muscular dystrophy type 1A (MDC1A), one of neuromuscular disorders, usually appears at birth or infancy. It is mainly featured by hypotonia, myasthenia and amyotrophy. MDC1A is caused by loss-of-function mutations in *LAMA2*, which encodes for laminin-α2. To compensate for the loss of laminin-α2, Ronald D. Cohn and his colleagues used CRISPRa to upregulate *LAMA1*, which encodes laminin-α1 and is a structurally similar protein to laminin-α2. Upregulation of LAMA1 ameliorates muscle wasting and paralysis in the MDC1A mouse model and provides a novel mutation-independent approach for disease correction 60.

### Genetic liver disease

Hereditary tyrosinemia type I (HTI) patients with loss of function *FAH* mutations accumulate toxic metabolites that cause liver damage. CRISPR/Cas- mediated HDR has been used to correct *FAH^mut/mu^*^t^ in the HTI mouse model by hydrodynamic injection of plasmids encoding CRISPR/Cas components or by combined delivery of AAV carrying HDR template and sgRNA and of nanoparticles with Cas9 mRNA 61, 62. VanLith et al. transplanted edited hepatocytes with corrected FAH into recipient FAH-knockout mice and cured HTI mice 63. Song et al. have used ABE in an adult mouse model of HTI to correct a *FAH* point mutation 64. In addition to correcting *FAH,* several groups have knocked out hydroxyphenylpyruvate dioxygenase (HPD), which acts in the second step of tyrosine catabolism and is an upstream enzyme of FAH, to prevent toxic metabolite accumulation and treat HTI metabolic disease 65.

Patients with alpha-1 antitrypsin deficiency (AATD) develop liver disease due to a toxic gain-of- function mutant allele, as well as progressive lung disease due to the loss of AAT antiprotease function. CRISPR/Cas-mediated NHEJ has been used to disrupt mutant AAT to reduce the pathologic liver phenotype 66, while HDR has been used to correct an AAT point mutation 67.

### Congenital genetic lung disease

Congenital genetic lung diseases include cystic fibrosis and inherited surfactant protein (SP) syndromes 68. Monogenic lung diseases caused by mutations in SP genes of the pulmonary epithelium show perinatal lethal respiratory failure death or chronic diffuse lung disease with few therapeutic options. Using a CRISPR fluorescent reporter system, scientists precisely timed intra-amniotic delivery of CRISPR/Cas9 components into a prenatal mouse model with the human SP gene *SFTPC^I73T^* mutation to inactivate mutant *SFTPC^I73T^* gene through NHEJ. Prenatal gene editing in* SFTPC^I73T^* mutant mice rescued lung pathophysiology, improved lung development, and increased survival rate to 22.8%. For intra- amniotic delivery, the amniotic cavity of embryonic day 16 mouse fetus, in which fetal breathing movements are optimal for fetal lung editing, was injected. After prenatal CRISPR delivery, embryonic day 19 fetus achieved up to 32% SFTPC wild-type airway and alveolar epithelial cells in *SFTPC^I73T^* mice, rescued lung pathophysiology by immunohistology, improved lung development by reducing the synthesis of mis trafficked *SFTPC* mutant proprotein, and increased survival rate to 22.8% 69.

Cystic fibrosis is another life-threatening monogenic lung disease caused by mutations in CFTR gene 70. Researchers applied CRISPR to precisely corrected CFTR carrying homozygous F508 deletion (F508del) in exon 10 in the induced pluripotent stem cells (iPSC) separated from cystic fibrosis patients 71 and the overall correction efficiency is up to 90% using *piggyBac* transposase as selection marker. Xu group applied the electroporation of CRISPR/Cas RNP and achieved more than 20% correction rate in patient-derived iPSC cell line with F508del mutation 72. As expected, CRISPR-induced genetic correction leads to the recovery of CFTR function in airway epithelial cells or proximal lung organoids derived from iPSC.

### Genetic deafness

At least half of all cases of profound congenital deafness are caused by genetic mutations and genetically inherited. Approximately 120 deafness- associated genes have been identified, but few treatments are available to slow or reverse genetic deafness 73. Recently, David R. Liu's group employed cationic lipid-mediated *in vivo* delivery of Cas9-guide RNA complexes to disrupt the dominant deafness-associated allele in the humanized transmembrane channel-like 1 (Tmc1) Beethoven (Bth) mouse model and ameliorated the hearing loss in these animals 74. David P. Corey's group screened 14 Cas9/sgRNA combinations and identified that SaCas9-KKH/gRNA could specially and safely recognize mutant *Tmc1* but not wildtype allele *in vitro* and *in vivo*, which provides a strategy to efficiently and selectively disrupt the dominant single nucleotide mutation rather than the wild-type alleles 75.

## Overcoming limitations of CRISPR/Cas-based gene therapy

Extensive work is being done with CRISPR/Cas in disease research and recent reviews had summarized the advantages of CRISPR/Cas 76, 77. The safety and efficacy of CRISPR/Cas9-based gene therapies need to be evaluated and refined before these therapies are applied in patients 78. One of the common limitations for CRISPR/Cas is that not all the mutation locus harbors the PAM motif, which the target recognition relies on. Besides, the challenges for using CRISPR/Cas as gene therapy include editing at off-target genomic sites, delivery vehicle, immunogenicity, and DNA damage response.

### Off-target effects of CRISPR/Cas

Despite significant advances in understanding the CRISPR/Cas9 system, concerns remain regarding off-target effects. Indeed, several groups found a tradeoff between activity and specificity of CRISPR/ Cas9, identifying off-target DNA cleavage by genome wide deep sequencing technique 79-81. Moreover, CBEs and ABEs cause transcriptome-wide off-target RNA editing 82, 83. Thus, unwanted off targets are concerns for the application of CRISPR. However, off-target effects can be reduced with sgRNA selection and optimization. Also, verification of *in vivo* off-targets (VIVO) can be used for defining and quantifying off-target editing of nucleases in whole organisms 84. The recently developed anti-CRISPR proteins could conditionally control the activity of the CRISPR system 85-88, which may show the potential in reducing off-target effects. The development of more sensitive methods is necessary for detecting off-target editing at both genome and transcriptome levels.

### *In vivo* delivery of CRISPR/Cas

AAV is the most widely used *in vivo* delivery of CRISPR/Cas. However, AAV has a limited packaging capacity, hindering all-in-one delivery of CRISPR/ Cas components, in particular larger Cas-derived base editor and prime editor. This has led to continued development of smaller Cas9 orthologues like SaCas9 13. For instance, saCas9 or NmeCas9 and sgRNA have been combined into a single AAV vector for inducing indels to correct disease. For disease correction by HDR or base editors, dual AAV or split AAV vectors can be used to circumvent packaging size limitations 89, 90. A disadvantage of such an approach is the requirement of uptake and expression of both AAV vectors into the same cell at roughly the same time to ensure intracellular Cas9:sgRNA complex formation.

CRISPR/Cas components can also be delivered by non-viral methods, for instance, Cas9 mRNA and sgRNA can be delivered to mouse liver by nanoparticles 62. But the external and internal barriers for nanoparticles entering the cell and nucleus must be considered. Currently, nanoparticles carrying CRISPR/Cas components are largely applied to mice and delivered into liver. Because the liver contains fenestrated capillary endothelia. Further improvement of nanoparticle-based CRISPR/Cas components delivery systems is needed for other target tissues.

### Immune response stimulated by CRISPR/Cas

The application of CRISPR/Cas systems raises concerns over immunogenicity of the bacterially- derived Cas9 protein 91. In a recent study, Charlesworth et al. demonstrated that anti-Cas9 responses are present in healthy human adults 92. In 34 human blood samples, anti-Cas9 IgG antibodies were detected against SaCas9 (79% of samples), and against SpCas9 (65% of samples). The immunogenicity of SpCas9 in healthy humans has been reported by Michael's group. Specifically, they found that high prevalence of effector T cells towards SpCas9 exist prior to the delivery of SpCas9 93. This issue will need to be addressed in the clinical applications of CRISPR/Cas.

### DNA damage response activated by CRISPR/Cas

In CRISPR/Cas gene editing via NHEJ and HDR, DSBs are generated at the target sites. DBS- based repair activates a p53-dependent DNA damage response and induces transient cell cycle arrest, leading to a decrease in efficiency of template- mediated precision genome editing 94. In human pluripotent stem cells, p53-deficient cells are more susceptible to CRISPR-mediated modification 95. These findings suggest that, during clinical trials, CRISPR-engineered cells or organs in patients should be monitored for p53 function. To avoid DSB triggered p53-mediated response, base editors (ABE and CBE) and prime editors can be applied for precision gene editing-mediated target gene correction.

## Conclusion and perspectives of using CRISPR/Cas in the clinic

CRISPR/Cas has already shown great potential in generating disease models and correcting monogenic disease mutations. The CRISPR disease models can accelerate the discovery and development of drug targets. In addition to the widely used type II CRISPR/Cas systems, continued discovery and development of CRISPR systems from prokaryotic species has generated new technologies. For example, DN1S-SpCas9 fusion protein blocks local NHEJ events and increases HDR frequency 96. Moreover, Cas13a-based RNA-targeting tools enable RNA changes that are temporally and spatially controllable, and will broaden and facilitate the application of RNA therapy in human diseases. Before the application of CRISPR for human disease correction, efforts are needed to optimize and maximize the editing efficiency as well as minimize off-targets and develop novel tools to specifically deliver the CRISPR components to the target tissue for gene editing 97, 98. As CRISPR/Cas-based gene therapy enters clinical trials (**Table 3**), this technology holds great potential for treating genetic diseases particularly for the present incurable ones and enhancing cell therapies.

## Figures and Tables

**Table 1:** Animal diseases models generated by CRISPR listed in this review.

Corresponding human disease	Targeted gene	Subsrate	Stragegy	Author, year, (Refs)
Duchenne muscular dystrophy (DMD)	*DMD*	Human rhabdomyosarcoma cell line	NHEJ-mediated exon removal	Shimo et al, 2018,(31)
Aniridia-related keratopathy (ARK)	*PAX6*	Human limbal epithelial cells	NHEJ-mediated mutation	Roux et al, 2018, (32)
Osteogenesis Imperfecta (OI)	*COL1A1*	Human MCRIi001-A iPSCs line	NHEJ-mediated a single base insertion	Far et al, 2019, (33)
X-linked adrenoleukodystrophy (X-ALD)	*ABCD1* & *ABCD2*	Murine BV-2 immortalized cell line	NHEJ-mediated gene deletion	Raas et al, 2019, (34)
Alzheimer's disease	*APPS* & *PSEN1M1*	Human and Mouse IPS Cell line	HDR-mediated mutation	Paquet et al, 2016, (35)
Duchenne muscular dystrophy (DMD)	*DMD*	Mouse	NHEJ-mediated exon removal	Egorova et al, 2019, (36)
Atherosclerosis	*LDLR*	Mouse liver	NHEJ-mediated gene deletion	Jarrett et al, 2018, (37)
Obesity (ob/ob) and diabetes (db/db)	*LEP* & *LEPR*	Mouse	NHEJ-mediated gene deletion	Roh et al, 2018, (38)
Resistance to thyroid hormone due to THRA mutation (RTHα)	*THRA*	Mouse	HDR-mediated mutation	Markossian et al, 2017, (39)
Alzheimer's disease (AD) and frontotemporal dementia (FTD)	*MAPT*	Mouse	NHEJ-mediated exon removal	Tan et al, 2018, (40)
Ryanodine receptor type I (RYR1)-related myopathies (RYR1 RM)	*RYR1*	Mouse muscle	HDR-mediated mutation	Brennan et al, 2019, (41)
Cystic fibrosis (CF)	*CFTR*	Sheep	NHEJ-mediated gene deletion	Fan et al, 2018, (42)
Diabetes mellitus (DM)	*PAX4*	Rabbit	NHEJ-mediated gene deletion	Xu et al, 2018, (43)
Huntington's disease (HD)	*HTT*	Pig	HDR-mediated exon fragments insertion	Yan et al, 2018, (44)
Autosomal recessive juvenile parkinsonism	*PINK1*	Monkey	NHEJ-mediated gene deletion	Yang et al, 2019, (45)

**Table 2:** Preclinical CRISPR Therapy in disease models listed in this review.

Diseases	Target (Gene accession number)	Animal model or substrate	Delivery System	Strategy	Outcome	Author, year, (Refs)
β-thalassemia	*HBB* (NC_000011.10)	CD34+ HSPCs of β-thalassemia patients	RNP; electroporation	NHEJ-mediated mRNA splicing	93.0% indel frequency (SpCas9)	Xu et al, 2019 (50)
Hemoglobinopathies	BCL11A erythroidenhancer (NC_000002.12)	CD34+ HSPCs from sickle cell disease patient	RNP; electroporation	NHEJ-mediated enhancer disruption	54.6% reduction of BCL11A expression	Wu et al, 2019, (52)
Leber congenital amaurosis type 10	CEP290 (NC_000012.12 )	HuCEP290 IVS26 KI mouse eye	AAV; subretinal injection	NHEJ-mediated aberrant splicing	~ 60% editing rates in mice	Maeder et al, 2019, (53)
Duchenne muscular dystrophy (DMD)	Dmd (NC_000086.7)	mdx mice muscle	AAV; intramuscular injection (IM), retro- orbital injection (RO) and intraperitoneal injection (IP)	NHEJ-mediated mutant exon 23 skipping	~52% of WT (IP) , ~71% of WT (RO), and ~70% of WT (IM) Dystrophin protein levels	Long et al, 2016, (55)
Duchenne muscular dystrophy (DMD)	Dmd (NC_000086.7)	mdx mice muscle	AAV; intramuscular injection	NHEJ-mediated mutant exon 23 skipping	~2% of all alleles from the whole muscle lysate	Nelson et al, 2016, (56)
Duchenne muscular dystrophy (DMD)	Dmd (NC_000086.7)	mdx mice muscle	AAV; intraperitoneal injection	NHEJ-mediated mutant exon 23 skipping	24-47% of total Dmd mRNA in cells including exon23 deletion	Tabebordbar et al, 2016, (57)
Congenital muscular dystrophy type 1A (MDC1A)	Lama1 (NC_000083.6 )	dy^2j^/dy^2j^ mouse	AAV; intramuscular or tail vein injection	CRISPR activator mediated gene upregulation	3.6-fold upregulation of Lama1	Kemaladewi et al, 2019, (60)
Hereditary tyrosinemia type I (HTI)	*FAH* (NC_000073.6)	FAH*^mut/mut^* mouse liver	AAV combined with lipid nanoparticles; intravenous injection	HDR-mediated point mutation correction	~0.8% initial correction rate in total liver DNA; more than 6% FAH+ hepatocytes	Yin et al, 2016, (62)
Hereditary tyrosinemia type I (HTI)	*FAH* (NC_000073.6)	FAH*^mut/mut^* mouse hepatocytes	AAV; transplantation	HDR-mediated point mutation correction	2.6% alleles were correted	VanLith et al, 2019, (63)
Hereditary tyrosinaemiatype I (HTI)	*FAH* (NC_000073.6)	FAH*^mut/mut^* mouse liver	plasmids; hydrodynamic tail-vein injection	Adenine base editor mediated point mutation correction	~0.3% initial correction rate in liver, ~4% FAH+ hepatocytes	Song et al, 2019, (64)
α1-antitrypsin deficiency (AATD)	AAT (NC_000078.6 )	PiZ mouse liver	AAV; intravenous injection	NHEJ-mediated mutant AAT disruption	~30% idel frequency	Bjursell et al, 2018, (66)
α1-antitrypsin deficiency (AATD)	AAT (NC_000078.6 )	PiZ mouse liver	AAV; intravenous injection	HdR-mediated point mutation correction	~2% correction rate in liver	Song et al, 2018, (67)
Perinatal Lethal Respiratory Failure	*SFTPC* ( NC_000080.6)	SFTPC^I73T^; R26^mTmG/+^ mouse fetus lung	adeno virus; intra-amniotic delivery	NHEJ-mediated mutant *SFTPC* disruption	~20% editing in the lung epithelium of fetuses	Alapati et al, 2019, (69)
Genetic Deafness	Tmc1 (NC_000085.6)	Beethoven (Bth)mouse ear	AAV; Inner-ear injections	NHEJ-mediated mutant Tmc allele disruption	2.2% indel frequencies at 55 days after injection; 24% decrease in Bth mRNA	György et al, 2019, (75)

**Table 3:** CRISPR clinical trials for inherited diseases listed in this review.

Disease	Study title	Strategy	Study phase	Study type	Participants (No., Age)	Company	NCT Number	Website
**Transfusion- Dependent β-thalassemia**	A Safety and Efficacy Study Evaluating CTX001 in Subjects With Transfusion-Dependent β-Thalassemia	CTX001	Phase 1Phase 2	Interventional	45 patients, ≥18 and ≤35 years of age	Vertex Pharmaceuticals Incorporated & CRISPR Therapeutics	NCT03655678	https://clinicaltrials.gov/ct2/show/NCT03655678
**Sickle Cell Disease**	A Safety and Efficacy Study Evaluating CTX001 in Subjects With Severe Sickle Cell Disease	CTX001	Phase 1Phase 2	Interventional	45 patients, ≥18 and ≤35 years of age	Vertex Pharmaceuticals Incorporated & CRISPR Therapeutics	NCT03745287	https://clinicaltrials.gov/ct2/show/NCT03745287
**β-thalassemia**	iHSCs With the Gene Correction of HBB Intervent Subjests With β-thalassemia Mutations	HBB HSC-01	Early Phase 1	Interventional	12 patients, ≥ 2 and ≤ 60 years of age	Allife Medical Science & Technology Co., Ltd.	NCT03728322	https://clinicaltrials.gov/ct2/show/NCT03728322
**Leber congenital amaurosis LCA10**	Single Ascending Dose Study in Participants With LCA10	AGN-151587	Phase 1Phase 2	Interventional	18 patients, ≥ 3 Years	Allergan & Editas Medicine, Inc.	NCT03872479	https://clinicaltrials.gov/ct2/show/NCT03872479

Data from https://clinicaltrials.gov/

## Abbreviations

ZFN: zinc finger nucleases
TALENS: transcription activator-like effector nucleases
CRISPR: clustered regularly interspaced short palindromic repeats
Cas: CRISPR-associated nuclease
SpCas9: Cas nuclease comes from *Streptococcus pyogenes*
sgRNA: single-guide RNA
PAM: protospacer adjacent motif
DSB: double-strand break
NHEJ: non- homologous end joining
HDR: homology-directed repair
indels: insertions and deletions
CRISPRa: CRISPR activators
CRISPRi: CRISPR inhibitors
AAV: adeno-associated virus
CBEs: cytidine base editors
ABEs: adenine base editors
PEs: prime editors
DMD: duchenne muscular dystrophy
HTI: hereditary tyrosinemia type I
AAV: adeno-associated virus

## References

[1] High KA, Roncarolo MG (2019). Gene Therapy. N Engl J Med.

[2] Doudna JA, Charpentier E (2014). The new frontier of genome engineering with CRISPR-Cas9. Science.

[3] Cox DB, Platt RJ, Zhang F (2015). Therapeutic genome editing: prospects and challenges. Nat Med.

[4] Yin H, Xue W, Anderson DG (2019). CRISPR-Cas: a tool for cancer research and therapeutics. Nat Rev Clin Oncol.

[5] Fellmann C, Gowen BG, Lin PC, Doudna JA, Corn JE (2017). Cornerstones of CRISPR-Cas in drug discovery and therapy. Nat Rev Drug Discov.

[6] Mojica FJ, Diez-Villasenor C, Garcia-Martinez J, Soria E (2005). Intervening sequences of regularly spaced prokaryotic repeats derive from foreign genetic elements. J Mol Evol.

[7] Gasiunas G, Barrangou R, Horvath P, Siksnys V (2012). Cas9-crRNA ribonucleoprotein complex mediates specific DNA cleavage for adaptive immunity in bacteria. Proc Natl Acad Sci U S A.

[8] Marraffini LA, Sontheimer EJ (2010). CRISPR interference: RNA-directed adaptive immunity in bacteria and archaea. Nat Rev Genet.

[9] Cong L, Ran FA, Cox D, Lin S, Barretto R, Habib N (2013). Multiplex genome engineering using CRISPR/Cas systems. Science.

[10] Gaj T, Gersbach CA, Barbas CF 3rd (2013). ZFN, TALEN, and CRISPR/Cas-based methods for genome engineering. Trends Biotechnol.

[11] Lieber MR (2010). The mechanism of double-strand DNA break repair by the nonhomologous DNA end-joining pathway. Annu Rev Biochem.

[12] Chiruvella KK, Liang Z, Wilson TE (2013). Repair of double-strand breaks by end joining. Cold Spring Harb Perspect Biol.

[13] Ran FA, Cong L, Yan WX, Scott DA, Gootenberg JS, Kriz AJ (2015). In vivo genome editing using Staphylococcus aureus Cas9. Nature.

[14] Edraki A, Mir A, Ibraheim R, Gainetdinov I, Yoon Y, Song CQ (2019). A Compact, High-Accuracy Cas9 with a Dinucleotide PAM for In Vivo Genome Editing. Mol Cell.

[15] Kampmann M (2018). CRISPRi and CRISPRa Screens in Mammalian Cells for Precision Biology and Medicine. ACS Chem Biol.

[16] Chen B, Gilbert LA, Cimini BA, Schnitzbauer J, Zhang W, Li GW (2013). Dynamic imaging of genomic loci in living human cells by an optimized CRISPR/Cas system. Cell.

[17] Wu X, Mao S, Ying Y, Krueger CJ, Chen AK (2019). Progress and Challenges for Live-cell Imaging of Genomic Loci Using CRISPR-based Platforms. Genomics Proteomics Bioinformatics.

[18] Chen B, Zou W, Xu H, Liang Y, Huang B (2018). Efficient labeling and imaging of protein-coding genes in living cells using CRISPR-Tag. Nat Commun.

[19] Anzalone AV, Randolph PB, Davis JR, Sousa AA, Koblan LW, Levy JM (2019). Search-and-replace genome editing without double-strand breaks or donor DNA. Nature.

[20] Cox DBT, Gootenberg JS, Abudayyeh OO, Franklin B, Kellner MJ, Joung J (2017). RNA editing with CRISPR-Cas13. Science.

[21] Abudayyeh OO, Gootenberg JS, Franklin B, Koob J, Kellner MJ, Ladha A (2019). A cytosine deaminase for programmable single-base RNA editing. Science.

[22] Oakes BL, Fellmann C, Rishi H, Taylor KL, Ren SM, Nadler DC (2019). CRISPR-Cas9 Circular Permutants as Programmable Scaffolds for Genome Modification. Cell.

[23] Yuan J, Ma Y, Huang T, Chen Y, Peng Y, Li B (2018). Genetic Modulation of RNA Splicing with a CRISPR-Guided Cytidine Deaminase. Mol Cell.

[24] Abudayyeh OO, Gootenberg JS, Essletzbichler P, Han S, Joung J, Belanto JJ (2017). RNA targeting with CRISPR-Cas13. Nature.

[25] Xie N, Zhou Y, Sun Q, Tang B (2018). Novel Epigenetic Techniques Provided by the CRISPR/Cas9 System. Stem Cells Int.

[26] Papasavva P, Kleanthous M, Lederer CW (2019). Rare Opportunities: CRISPR/Cas-Based Therapy Development for Rare Genetic Diseases. Mol Diagn Ther.

[27] Kennedy EM, Cullen BR (2017). Gene Editing: A New Tool for Viral Disease. Annu Rev Med.

[28] Huang CH, Lee KC, Doudna JA (2018). Applications of CRISPR-Cas Enzymes in Cancer Therapeutics and Detection. Trends Cancer.

[29] Xiong X, Chen M, Lim WA, Zhao D, Qi LS (2016). CRISPR/Cas9 for Human Genome Engineering and Disease Research. Annu Rev Genomics Hum Genet.

[30] Ferdosi SR, Ewaisha R, Moghadam F, Krishna S, Park JG, Ebrahimkhani MR (2019). Multifunctional CRISPR-Cas9 with engineered immunosilenced human T cell epitopes. Nat Commun.

[31] Shimo T, Hosoki K, Nakatsuji Y, Yokota T, Obika S (2018). A novel human muscle cell model of Duchenne muscular dystrophy created by CRISPR/Cas9 and evaluation of antisense-mediated exon skipping. J Hum Genet.

[32] Roux LN, Petit I, Domart R, Concordet JP, Qu J, Zhou H (2018). Modeling of Aniridia-Related Keratopathy by CRISPR/Cas9 Genome Editing of Human Limbal Epithelial Cells and Rescue by Recombinant PAX6 Protein. Stem Cells.

[33] Hosseini Far H, Patria YN, Motazedian A, Elefanty AG, Stanley EG, Lamande SR (2019). Generation of a heterozygous COL1A1 (c.3969_3970insT) osteogenesis imperfecta mutation human iPSC line, MCRIi001-A-1, using CRISPR/Cas9 editing. Stem Cell Res.

[34] Raas Q, Gondcaille C, Hamon Y, Leoni V, Caccia C, Menetrier F (2019). CRISPR/Cas9-mediated knockout of Abcd1 and Abcd2 genes in BV-2 cells: novel microglial models for X-linked Adrenoleukodystrophy. Biochim Biophys Acta Mol Cell Biol Lipids.

[35] Paquet D, Kwart D, Chen A, Sproul A, Jacob S, Teo S (2016). Efficient introduction of specific homozygous and heterozygous mutations using CRISPR/Cas9. Nature.

[36] Egorova TV, Zotova ED, Reshetov DA, Polikarpova AV, Vassilieva SG, Vlodavets DV (2019). CRISPR/Cas9-generated mouse model of Duchenne muscular dystrophy recapitulating a newly identified large 430 kb deletion in the human DMD gene. Dis Model Mech.

[37] Jarrett KE, Lee C, De Giorgi M, Hurley A, Gillard BK, Doerfler AM (2018). Somatic Editing of Ldlr With Adeno-Associated Viral-CRISPR Is an Efficient Tool for Atherosclerosis Research. Arterioscler Thromb Vasc Biol.

[38] Roh JI, Lee J, Park SU, Kang YS, Lee J, Oh AR (2018). CRISPR-Cas9-mediated generation of obese and diabetic mouse models. Exp Anim.

[39] Markossian S, Guyot R, Richard S, Teixeira M, Aguilera N, Bouchet M (2018). CRISPR/Cas9 Editing of the Mouse Thra Gene Produces Models with Variable Resistance to Thyroid Hormone. Thyroid.

[40] Tan DCS, Yao S, Ittner A, Bertz J, Ke YD, Ittner LM (2018). Generation of a New Tau Knockout (tauDeltaex1) Line Using CRISPR/Cas9 Genome Editing in Mice. J Alzheimers Dis.

[41] Brennan S, Garcia-Castaneda M, Michelucci A, Sabha N, Malik S, Groom L (2019). Mouse model of severe recessive RYR1-related myopathy. Hum Mol Genet.

[42] Fan Z, Perisse IV, Cotton CU, Regouski M, Meng Q, Domb C (2018). A sheep model of cystic fibrosis generated by CRISPR/Cas9 disruption of the CFTR gene. JCI Insight.

[43] Xu Y, Wang Y, Song Y, Deng J, Chen M, Ouyang H (2018). Generation and Phenotype Identification of PAX4 Gene Knockout Rabbit by CRISPR/Cas9 System. G3 (Bethesda).

[44] Yan S, Tu Z, Liu Z, Fan N, Yang H, Yang S (2018). A Huntingtin Knockin Pig Model Recapitulates Features of Selective Neurodegeneration in Huntington's Disease. Cell.

[45] Yang W, Li S, Li XJ (2019). A CRISPR monkey model unravels a unique function of PINK1 in primate brains. Mol Neurodegener.

[46] Landrum MJ, Lee JM, Benson M, Brown G, Chao C, Chitipiralla S (2016). ClinVar: public archive of interpretations of clinically relevant variants. Nucleic Acids Res.

[47] Thein SL (2013). The molecular basis of beta-thalassemia. Cold Spring Harb Perspect Med.

[48] Heywood JD, Karon M, Weissman S (1964). Amino Acids: Incorporation into α- and β-Chains of Hemoglobin by Normal and Thalassemic Reticulocytes. Science.

[49] Telen MJ, Malik P, Vercellotti GM (2019). Therapeutic strategies for sickle cell disease: towards a multi-agent approach. Nat Rev Drug Discov.

[50] Xu S, Luk K, Yao Q, Shen AH, Zeng J, Wu Y (2019). Editing aberrant splice sites efficiently restores β-globin expression in β-thalassemia. Blood.

[51] Canver MC, Smith EC, Sher F, Pinello L, Sanjana NE, Shalem O (2015). BCL11A enhancer dissection by Cas9-mediated in situ saturating mutagenesis. Nature.

[52] Wu Y, Zeng J, Roscoe BP, Liu P, Yao Q, Lazzarotto CR (2019). Highly efficient therapeutic gene editing of human hematopoietic stem cells. Nat Med.

[53] Maeder ML, Stefanidakis M, Wilson CJ, Baral R, Barrera LA, Bounoutas GS (2019). Development of a gene-editing approach to restore vision loss in Leber congenital amaurosis type 10. Nat Med.

[54] McCullough KT, Boye SL, Fajardo D, Calabro K, Peterson JJ, Strang CE (2019). Somatic Gene Editing of GUCY2D by AAV-CRISPR/Cas9 Alters Retinal Structure and Function in Mouse and Macaque. Hum Gene Ther.

[55] Long C, Amoasii L, Mireault AA, McAnally JR, Li H, Sanchez-Ortiz E (2016). Postnatal genome editing partially restores dystrophin expression in a mouse model of muscular dystrophy. Science.

[56] Nelson CE, Hakim CH, Ousterout DG, Thakore PI, Moreb EA, Castellanos Rivera RM (2016). In vivo genome editing improves muscle function in a mouse model of Duchenne muscular dystrophy. Science.

[57] Tabebordbar M, Zhu K, Cheng JKW, Chew WL, Widrick JJ, Yan WX (2016). In vivo gene editing in dystrophic mouse muscle and muscle stem cells. Science.

[58] Amoasii L, Hildyard JCW, Li H, Sanchez-Ortiz E, Mireault A, Caballero D (2018). Gene editing restores dystrophin expression in a canine model of Duchenne muscular dystrophy. Science.

[59] Ryu SM, Koo T, Kim K, Lim K, Baek G, Kim ST (2018). Adenine base editing in mouse embryos and an adult mouse model of Duchenne muscular dystrophy. Nat Biotechnol.

[60] Kemaladewi DU, Bassi PS, Erwood S, Al-Basha D, Gawlik KI, Lindsay K (2019). A mutation-independent approach for muscular dystrophy via upregulation of a modifier gene. Nature.

[61] Yin H, Xue W, Chen S, Bogorad RL, Benedetti E, Grompe M (2014). Genome editing with Cas9 in adult mice corrects a disease mutation and phenotype. Nat Biotechnol.

[62] Yin H, Song CQ, Dorkin JR, Zhu LJ, Li Y, Wu Q (2016). Therapeutic genome editing by combined viral and non-viral delivery of CRISPR system components in vivo. Nat Biotechnol.

[63] VanLith CJ, Guthman RM, Nicolas CT, Allen KL, Liu Y, Chilton JA (2019). Ex Vivo Hepatocyte Reprograming Promotes Homology-Directed DNA Repair to Correct Metabolic Disease in Mice After Transplantation. Hepatol Commun.

[64] Song C-Q, Jiang T, Richter M, Rhym LH, Koblan LW, Zafra MP (2020). Adenine base editing in an adult mouse model of tyrosinaemia. Nat Biomed Eng.

[65] Pankowicz FP, Barzi M, Legras X, Hubert L, Mi T, Tomolonis JA (2016). Reprogramming metabolic pathways in vivo with CRISPR/Cas9 genome editing to treat hereditary tyrosinaemia. Nat Commun.

[66] Bjursell M, Porritt MJ, Ericson E, Taheri-Ghahfarokhi A, Clausen M, Magnusson L (2018). Therapeutic Genome Editing With CRISPR/Cas9 in a Humanized Mouse Model Ameliorates alpha1-antitrypsin Deficiency Phenotype. EBioMedicine.

[67] Song CQ, Wang D, Jiang T, O'Connor K, Tang Q, Cai L (2018). In Vivo Genome Editing Partially Restores Alpha1-Antitrypsin in a Murine Model of AAT Deficiency. Hum Gene Ther.

[68] Whitsett JA, Wert SE, Weaver TE (2010). Alveolar surfactant homeostasis and the pathogenesis of pulmonary disease. Annu Rev Med.

[69] Alapati D, Zacharias WJ, Hartman HA, Rossidis AC, Stratigis JD, Ahn NJ (2019). In utero gene editing for monogenic lung disease. Sci Transl Med.

[70] Rogan MP, Stoltz DA, Hornick DB (2011). Cystic Fibrosis Transmembrane Conductance Regulator Intracellular Processing, Trafficking, and Opportunities for Mutation-Specific Treatment. CHEST.

[71] Firth AL, Menon T, Parker GS, Qualls SJ, Lewis BM, Ke E (2015). Functional Gene Correction for Cystic Fibrosis in Lung Epithelial Cells Generated from Patient iPSCs. Cell Rep.

[72] Ruan J, Hirai H, Yang D, Ma L, Hou X, Jiang H (2019). Efficient Gene Editing at Major CFTR Mutation Loci. Mol Ther Nucleic Acids.

[73] Muller U, Barr-Gillespie PG (2015). New treatment options for hearing loss. Nat Rev Drug Discov.

[74] Gao X, Tao Y, Lamas V, Huang M, Yeh WH, Pan B (2018). Treatment of autosomal dominant hearing loss by in vivo delivery of genome editing agents. Nature.

[75] Gyorgy B, Nist-Lund C, Pan B, Asai Y, Karavitaki KD, Kleinstiver BP (2019). Allele-specific gene editing prevents deafness in a model of dominant progressive hearing loss. Nat Med.

[76] Molla KA, Yang Y (2019). CRISPR/Cas-Mediated Base Editing: Technical Considerations and Practical Applications. Trends Biotechnol.

[77] Knott GJ, Doudna JA (2018). CRISPR-Cas guides the future of genetic engineering. Science.

[78] Rich K, Terry SF (2018). CRISPR-Cas9: New Heights, New Hesitations. Genet Test Mol Biomarkers.

[79] Park SH, Lee CM, Dever DP, Davis TH, Camarena J, Srifa W (2019). Highly efficient editing of the beta-globin gene in patient-derived hematopoietic stem and progenitor cells to treat sickle cell disease. Nucleic Acids Res.

[80] Wang D, Zhang C, Wang B, Li B, Wang Q, Liu D (2019). Optimized CRISPR guide RNA design for two high-fidelity Cas9 variants by deep learning. Nat Commun.

[81] Ikeda A, Fujii W, Sugiura K, Naito K (2019). High-fidelity endonuclease variant HypaCas9 facilitates accurate allele-specific gene modification in mouse zygotes. Commun Biol.

[82] Grunewald J, Zhou R, Garcia SP, Iyer S, Lareau CA, Aryee MJ (2019). Transcriptome-wide off-target RNA editing induced by CRISPR-guided DNA base editors. Nature.

[83] Zhou C, Sun Y, Yan R, Liu Y, Zuo E, Gu C (2019). Off-target RNA mutation induced by DNA base editing and its elimination by mutagenesis. Nature.

[84] Akcakaya P, Bobbin ML, Guo JA, Malagon-Lopez J, Clement K, Garcia SP (2018). In vivo CRISPR editing with no detectable genome-wide off-target mutations. Nature.

[85] Nakamura M, Srinivasan P, Chavez M, Carter MA, Dominguez AA, La Russa M (2019). Anti-CRISPR-mediated control of gene editing and synthetic circuits in eukaryotic cells. Nat Commun.

[86] Zhang F, Song G, Tian Y (2019). Anti-CRISPRs: The natural inhibitors for CRISPR-Cas systems. Animal Model Exp Med.

[87] Rauch BJ, Silvis MR, Hultquist JF, Waters CS, McGregor MJ, Krogan NJ (2017). Inhibition of CRISPR-Cas9 with Bacteriophage Proteins. Cell.

[88] Pawluk A, Amrani N, Zhang Y, Garcia B, Hidalgo-Reyes Y, Lee J (2016). Naturally Occurring Off-Switches for CRISPR-Cas9. Cell.

[89] Hirsch ML, Agbandje-McKenna M, Samulski RJ (2010). Little vector, big gene transduction: fragmented genome reassembly of adeno-associated virus. Mol Ther.

[90] Hirsch ML, Wolf SJ, Samulski RJ (2016). Delivering Transgenic DNA Exceeding the Carrying Capacity of AAV Vectors. Methods Mol Biol.

[91] Wang D, Mou H, Li S, Li Y, Hough S, Tran K (2015). Adenovirus-Mediated Somatic Genome Editing of Pten by CRISPR/Cas9 in Mouse Liver in Spite of Cas9-Specific Immune Responses. Hum Gene Ther.

[92] Charlesworth CT, Deshpande PS, Dever DP, Camarena J, Lemgart VT, Cromer MK (2019). Identification of preexisting adaptive immunity to Cas9 proteins in humans. Nat Med.

[93] Wagner DL, Amini L, Wendering DJ, Burkhardt LM, Akyuz L, Reinke P (2019). High prevalence of Streptococcus pyogenes Cas9-reactive T cells within the adult human population. Nat Med.

[94] Haapaniemi E, Botla S, Persson J, Schmierer B, Taipale J (2018). CRISPR-Cas9 genome editing induces a p53-mediated DNA damage response. Nat Med.

[95] Ihry RJ, Worringer KA, Salick MR, Frias E, Ho D, Theriault K (2018). p53 inhibits CRISPR-Cas9 engineering in human pluripotent stem cells. Nat Med.

[96] Jayavaradhan R, Pillis DM, Goodman M, Zhang F, Zhang Y, Andreassen PR (2019). CRISPR-Cas9 fusion to dominant-negative 53BP1 enhances HDR and inhibits NHEJ specifically at Cas9 target sites. Nat Commun.

[97] Ledford H (2019). CRISPR babies: when will the world be ready?. Nature.

[98] Chaterji S, Ahn EH, Kim D-H (2017). CRISPR Genome Engineering for Human Pluripotent Stem Cell Research. Theranostics.

